# Miscellanies

**Published:** 1840-04-01

**Authors:** 


### MISCELLANIES.

### PREVITE'S PREPARED CEYLON MOSS.

We have been favoured with samples of this article, prepared with great care by Mr. P. and capable of forming a delicate and nutritious jelly by half an hour's boiling. It becomes sufficiently firm in ten or fifteen minutes after being taken from the fire, and is nearly tasteless. The analysis of it by Dr. O'Shaughnessy, of Calcutta, shews the following composition.

| | |
|---|---|
| Vegetable jelly | 54.50 |
| Fine starch | 15.0 |
| Wax | a trace |
| Ligneous fibre | 18.0 |
| Gum | 4.0 |
| Sulphate and muriate of soda | 6.5 |
| Sulphate and phosphate of lime | 1.0 |
| Iron | a trace |
| | 98.55 |

As far as we have yet observed, this will prove a valuable dietetic article in the sick room.

### VACCINATION.

Lord Ellenborough has brought in a Bill, entitled "An Act to extend the Practice of Vaccination," which was read for the first time in the House of Lords on March 12th. We copy the main clauses.

1. Whereas it is expedient to extend the practice of vaccination, be it therefore enacted, &c. that from and after the passing of this Act it shall be lawful for the guardians of every Poor-Law Union in England and Wales, and they are hereby directed, to contract with the medical officers of their several Unions respectively for the vaccination of all children who may be brought to them for that purpose.

2. That such guardians shall, after consultation with such medical officers, from time to time appoint and give due notice of the appointment of such, and to so many convenient places and times as to them may seem fit, at which such medical officers shall attend to vaccinate all children who may be brought to them for that purpose; provided always that not more than three calendar months shall in any case elapse between the times at which such medical officers shall so attend.

3. That such medical officers shall make a report to the guardians of the several Poor-Law Unions in which they may act respectively on the next day of the meeting of such guardians after every such time so appointed as aforesaid for the vaccination of children, of the number of children then vaccinated, and from time to time shall make such further report with respect to the children so vaccinated, as the guardians of the several Poor-Law Unions, under the direction of the Poor-Law Commissioners, shall require.

5. That every such medical person shall give the like attendance and make the like reports as is and are hereinbefore required from the medical officer of any Union.

Lord Normanby has added a clause, which renders it penal for any non-medical person to inoculate the small-pox.

---

### Regulations respecting Assistant-Surgeons in the Navy.

By a recent regulation of the Lords of the Admiralty, an Assistant-Surgeon entering the Naval Service after the 1st Feb. is to be appointed by *an acting order*, to be confirmed on the expiration of *one year* from the original date, provided he produces a favourable testimonial of his moral character and attention to his duty, and of his strict and humane attention in the execution of his medical duties, and of his having made diligent use of the opportunities afforded him to improve his professional knowledge, both by study and practice. The certificate to be signed by the *Captain* and Surgeon; if at the hospital, by the *Captain Superintendant* and Physician.—*Naval and Military Gazette*, March 7, 1840.

---

### Mr. R. Alcock.

We learn that a distinguished honour has lately been conferred upon a member of the profession by the Queen of Spain. By Royal decree the insignia of Knight of the Royal Order of Charles III. has been bestowed upon Mr. Rutherford Alcock, for his services in Spain while Deputy Inspector-General of Hospitals in that country. This is the second most distinguished order in Spain, being ranked next in estimation to the Golden Fleece. We believe that Mr. Alcock is the third member of the medical profession in England who has obtained it. Dr. Hume, the Duke of Wellington's physician, received this honour at the close of the Peninsular war. According to a late history of distinguished European orders of chivalry conferred on British subjects, there are not more than thirty who have received this decoration.—*Medical Gazette, Jan.* 31, 1840.

---

### Montyon Prizes.

On a report made by M. Serres, in the name of the Commission charged with the examination of the papers sent in for the concours for the prizes in medicine and surgery on the Montyon foundation, the Academy (of Sciences) accorded gold medals of the value of 1500 francs to Dr. Bright of London, and to Drs. Martin-Solon, and Rayer, for their works on albuminuria or albuminous nephritis; a gold medal of the same value to M. Ricord for his treatise on syphilitic disease; and the sum of 1000 francs to M. Martin for the invention of an artificial leg. *Comptes Rendus*, Dec. 9, 1839.—*Ibid.*

---

### Retirement of Sir Benjamin Brodie from St. George's Hospital.

Sir Benjamin Brodie, after many years of most active and valuable service, has resigned the office of Surgeon to St. George's Hospital. We are glad to learn that his lectures to the pupils of the establishment are to be continued in the same manner as heretofore.—*Med. Gaz.* Jan. 17, 1840.

It is impossible not to appreciate the motives which have led to this resignation on the part of Sir Benjamin Brodie. The extent of his private practice rendered a regular and conscientious performance of his hospital duties impossible, or at all events incompatible with comfort and health. Under these circumstances, he preferred resigning a situation of honour and profit, to filling it inefficiently or doing the work by deputy.

To those who knew anything of Sir Benjamin Brodie, such feelings and such a step could excite little surprise. But the same spirit which could prompt the latter, will, we are confident, urge him on in the path of professional utility. His experience is still given to the pupils of the School of St. George's Hospital in the shape of lectures, and will not, we trust, be withheld from us all in that of distinct publications. There is no man at the present day who enjoys such opportunities for observation as Sir Benjamin Brodie has done, and there is no one, we will add, who possesses in a higher degree the faculty of turning that observation to account.

---

### Death of Mr. Copland Hutchison.

We regret very much to announce the death of this gentleman, which recently took place at Plymouth. Mr. Hutchison was the author of a work on Surgery of considerable value, as well as of vaiious papers in the Medico-Chirurgical Transactions.—*Ibid.*

